# Colchicine-free remission in familial Mediterranean fever: featuring a unique subset of the disease-a case control study

**DOI:** 10.1186/1750-1172-9-3

**Published:** 2014-01-09

**Authors:** Ilan Ben-Zvi, Tami Krichely-Vachdi, Olga Feld, Merav Lidar, Shaye Kivity, Avi Livneh

**Affiliations:** 1 Department of Medicine F and the Rheumatology unit, Sheba Medical Center, Tel-Hashomer, Israel; 2 Sackler School of Medicine, Tel Aviv University, Tel Aviv, Israel; 3 The Pinchas Borenstein Talpiot Medical Leadership Program 2012, Chaim Sheba, Medical Center, Tel-Hashomer, Israel

**Keywords:** Familial Mediterranean fever, Remission, Genetics, Demographics, Severity, Colchicine

## Abstract

**Background:**

To demonstrate and clinically, genetically and demographically characterize familial Mediterranean fever (FMF) patients, maintaining remission despite colchicine abstinence.

**Methods:**

FMF patients were screened for an endurance of prolonged remission (≥ 3 years), despite refraining from colchicine. Clinical, demographic and genetic parameters were collected. Data were compared with those of consecutive control FMF subjects, coming to the clinic for their periodic follow up examination.

**Results:**

Of 1000 patients screened over 5 years, 33 manifested colchicine-free remission. The mean duration of the remission period was 12.6 ± 8.1 years. Patients in the remission group had milder severity of FMF, compared to the control group (22 vs. 11 patients with mild disease, respectively, p = 0.003) and a longer diagnosis delay (21 ± 15.7 vs. 13.4 ± 13.5 years, respectively, p = 0.04). Patients experiencing remission suffered mostly of abdominal attacks, low rate of attacks in other sites and low rate of chronic and non-attack manifestations. When the disease resumed activity, it responded well to colchicine, despite using a lower dose, as compared to the control subjects (p < 0.001). None of the patients in this group was homozygous for the M694V mutation (p = 0.0008).

**Conclusions:**

Prolonged colchicine-free remission defines a rare and milder form of FMF with unique clinical, demographic, and molecular characteristics.

## Introduction

Familial Mediterranean fever (FMF) is a genetic, autoinflammatory disease, characterized by acute episodes of serosal and cutaneous inflammation, expressed with pain, fever, neutrophilia and intense acute-phase response, caused by activation of the innate immune system. Between the episodes of inflammation, patients are usually asymptomatic, but subclinical inflammation may persist [1], reflected by continuously elevated acute phase reactants and the development of AA amyloidosis in some patients.

The gene associated with FMF, the MEditerranean FeVer gene (*MEFV*) [2,3], was cloned from chromosome 16, and its protein product, pyrin, was shown to be part of the inflammasome complex that modify inflammation [4]. To date, over 100 disease-associated MEFV alterations, mostly missense mutations*,* have been identified, many of which cluster in exon 10 of the gene [5].

Short lived bouts of fever, peritonitis, pleuritis, arthritis/arthralgia and erysipelas-like erythema, culminating in spontaneous remission and recurring at a rate spanning between few to multiple attacks per year, are the cardinal clinical manifestations of FMF, its hallmark, but also the basis of its phenotype diversity. Non-uniformity of the FMF manifestations is a major obstacle for understanding of the pathogenesis, biochemical triggers and varying severity of the disease.

Structured scores, rating disease activity [6], or severity [7], using attack parameters (site, duration and frequency), are one way to classify and settle disease diversity. Using this approach, allocating severe phenotypes to certain genotypes, particularly to homozygous M694V, was noted [8]. However, a better understanding of the molecular basis of FMF may be derived from fine tuning, sorting out the disease to its clinical subsets.

To date, definition of separate phenotypes of FMF have been focused mainly on the severe end of the spectrum, particularly patients with active disease, not responding to colchicine, while the other end of the disease spectrum was scarcely accounted for. In the present report we set out to clinically, genetically and demographically characterize patients with FMF and low disease activity, specifically looking at patients who were or currently are free of attacks for a long time, despite being off colchicine.

## Methods

### Setting

The Israeli National Center for FMF at the Sheba Medical Center, Tel-Hashomer, is the largest center for FMF in Israel. Patients registered in the center are followed up regularly, at intervals of 6–12 months, and their demographic and genetic data are saved each visit on a digital file, which later may be processed and analyzed.

### Study group

All FMF patients, identified with prolonged remission were assigned to the study (remission group). They were identified during their clinic visit, from a cohort of about 1000 different FMF patients, coming to their regular periodic visit. Long-term remission was defined as a time interval of ≥ 3 years, without known clinical manifestations of FMF and without colchicine treatment, subsequent to a period of FMF activity. Patients were included whether the remission was still enduring at the time of clinic visit or has occurred in the past. The diagnosis of FMF for each patient was established according to widely accepted criteria [9].

### Control group

The remission group was compared to a similar size control group, consisting of consecutive, unselected and unmatched FMF patients, from the same cohort of 1000 FMF patients as above. Diagnosis of FMF was based on the same set of criteria. Unadjustment of the control group was determined to allow unrestricted comparison. To ensure random recruitment, subjects were assigned to the control group by another author (TKV), who was not aware of the study group features, prior to the recruitment of the control subjects.

### Study design

Once recruited, a questionnaire focusing on demographic, genetic, clinical and other parameters was completed, based on interview, physical examination and patients’ charts. The severity of the disease was determined, using an accepted FMF severity score [7]. The ethical committee of our institute (the Sheba Helsinki committee) approved the study protocol, and all patients involved signed an informed consent.

### Genetic analysis

*MEFV* mutations were obtained from patient charts. In case they were unavailable, mutational analysis of the five most common *MEFV* mutations (M694V, V726A, M680I, M694I and E148Q) was performed, using polymerase chain reaction with commercial kits and widely used methods, described elsewhere [10].

### Statistical analysis

Results are given as a mean ± standard deviation or proportions, as appropriate. Differences between the groups in discrete variables were evaluated by chi-square or Fisher’s exact test, according to sample size, as needed. Comparisons of continuous variables were evaluated by unpaired Student *t* test or Wilcoxon 2-sample test, as determined by cell size. Multivariate analysis to determine the parameters predicting long-term remission was performed, using the multiple logistic regression method, with variables reaching significant difference on univariate analysis. All p values are 2-sided. P values < 0.05 were considered significant.

## Results

**Figure 1:**
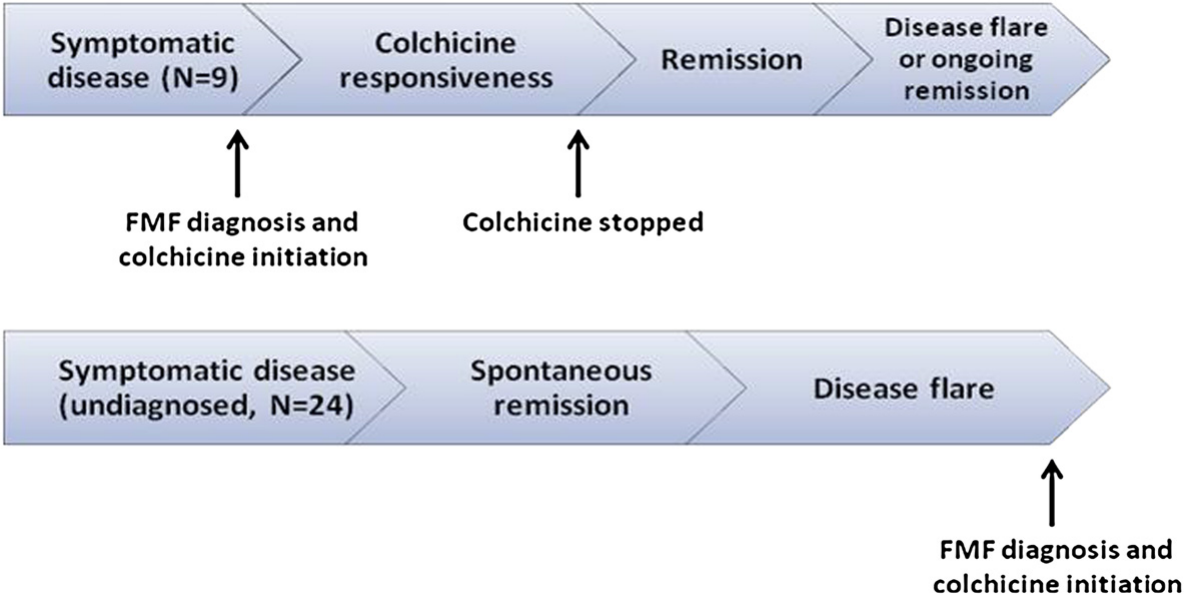
Linkage between colchicine-free remission and diagnosis of FMF*. *Colchicine free remission may occur following or prior to FMF diagnosis and onset of colchicine treatment.

In a cohort of 1000 FMF patients, we found 33 (19 males), who had long term remission despite being off colchicine (prevalence = 3.3%). The control group comprised 33 subjects (16 males, p = 0.31), recruited as described in the Methods section. Patients entered into remission of their FMF activity either after being diagnosed with FMF (9 patients), or prior to the diagnosis (24 patients, Figure 1). In the first scenario, patients presented with attacks, received a diagnosis of FMF and colchicine treatment and then, after a period without FMF activity under colchicine (for a mean of 16.4 years), they stopped colchicine upon their own decision, but nevertheless remission continued, despite being off colchicine. In the other scenario, patients experienced FMF attacks, for a mean of 9 years, without receiving a diagnosis of FMF or colchicine treatment. Then, FMF attacks have discontinued and patients entered remission (for a mean of 15.4 years). Only when the attacks resumed, a diagnosis of FMF was established and colchicine prescribed.

The mean age at remission onset was 25.2 ± 13.6 years. The mean duration of remission was 12.6 ± 8.1 years, but as some of the patients were still in remission at the end of the study, the final average duration of remission is even longer. Forty-eight percent of the patients in the remission group identified a presumed trigger for entering remission, the most frequent of which was abdominal operation (50%), in particular appendectomy. Other presumed triggers were nutritional change, a decrease in psychological stress, entering menopause, pregnancy and the use of contraceptives. Presumed triggers for disease flare after the period of remission was noted in 40% of the patients, the most frequent of which was enrollment to an obligatory army-service (30%). Other triggers were increased psychological stress, pregnancy or delivery and the use of contraceptive agents.

Table 1 presents the demographic characteristics of patients in the remission and control groups. Of note, while the mean age of disease onset is not significantly different, the mean age of diagnosis and hence the age of colchicine administration differ significantly between the 2 groups (p = 0.007). There were no significant differences in the ethnic origin or in the rate of family history of FMF between the 2 groups.

**Table 1:** Demographic characteristics of FMF patients with long term remission

**Variable**	**Remission group**	**Control**	**P value**
	**N = 33**	**N = 33**	
**Male gender (%)**	57.6	48.5	.311
**Age (Mean ± Std)**			
Age at study recruitment	46.82 ± 15.23	40.73 ± 13.77	.093
Age at disease onset	16.61 ± 12.92	12.79 ± 10.92	.199
Age at diagnosis	37.61 ± 18.29	26.21 ± 14.85	.007
Average delay in diagnosis (years)	21.00 ± 15.74	13.42 ± 13.58	.040
**Ethnicity (%)**			
N. Africa	21.2	36.4	.584
Iraq	18.2	12.1	
Greece/Turkey	12.1	3.0	
European	9.1	9.1	
Mixed	33.3	30.3	
Other	6.1	9.1	
**Family history (%)**	63.6	63.6	.601
**Family members with long-term remission**	3	0	0.5

The disease characteristics are shown in Table 2. There were significantly less patients in the remission group who experienced attacks of arthritis, pleuritis and erysipelas-like erythema, all of which are correlates of a more severe phenotype of FMF [7]. Indeed, patients in the remission group had a significantly lower severity score of their disease, compared to the control group (P = 0.003). The severity of the disease was even lower prior to the remission period, when 84% of patients had mild disease as compared to 63% after relapse of the disease (P = 0.04). The rate of individuals with abdominal attacks was also lower in the remission group, but has not reached statistical significance. Similarly, the rate of non-attack manifestations, such as proteinuria and anemia was also insignificantly lower in the remission group, except for exertional leg pain, which appeared to be significant (p = 0.03). All patients in the remission group, who relapsed and were placed on colchicine, responded very well to colchicine treatment while only 87.9% of the controls did so. Also, significantly more patients in the control group needed a colchicine dose of 2 mg/day or higher to stop or reduce the attacks. Finally, while off colchicine and still during their remission, all patients in the remission group had normal acute phase reactants (erythrocyte sedimentation rate or C– reactive protein) while 30% of the control group had increased levels of these parameters, despite being on therapeutic colchicine dose.

**Table 2:** Clinical characteristics of FMF patients with long-term remission

**Variable**	**Remission group**^**1**^	**Control**	**P value**
	**N = 33**	**N = 33**	
**FMF attacks (%)**			
Peritonitis	81.8	97.0	.052
Arthritis	24.2	48.5	.036
ELE	12.1	39.4	.011
Pleuritis	21.2	51.5	.010
**Non-attack manifestations (%)**			
Exertional leg-pain	18.2	42.4	.030
Splenomegaly	15.2	12.1	.500
Anemia	3.0	18.2	.052
Proteinuria or amyloidosis^**2**^	0	3.0	.500
Renal failure	0	6.1	.246
Dialysis	0	3.0	.500
**Treatment (%)**			
Good response to colchicine^**3**^	100.0	87.9	.057
Colchicine dose ≥ 2 mg/day^**4**^	9.1	51.5	<.001
**Severity score (%)**			
Mild	69.7	33.3	.003
Moderate or severe	30.3	66.7	
**Elevated APRs (%)**	0	30	

*ELE* Erysipelas like erythema. *APRs* Acute phase reactants (ESR or CRP). ^**1**^Over the entire course of their disease. ^**2**^Biopsy proven.^**3**^Defined as less than one attack per month while on colchicine treatment.^**4**^Adjusted according to patient’s response.

The genetic characteristics of the 2 groups are shown in Table 3. None of the patients in the remission group was homozygous to the M694V mutation, which is known to be associated with a more severe phenotype of the disease, while 32% of the patients in the control group were homozygous for this mutation (P = 0.0008). There were no significant differences in the carriage of other mutations between the two groups. Of note, the composition of genotypes in those with the longest remission (>10 years, 18 patients) was comparable to that in patients with shorter remission (13 patients, data not shown).

**Table 3:** Genetic characteristics of FMF patients with long-term remission

**Genotype**	**Remission group**	**Control**	**P value**
	**N = 33**	**N = 33**	
**w/M694V**	17 (51)	12 (35)	.158
**Heterozygous or homozygous to other mutations**	11 (34)	6 (19)	.130
V726A/V726A	3	2	
V726A/0	5	2	
V726A/E148Q	3	-	
V726A/M680I	-	1	
E148Q/0	-	1	
**Homozygous to M694V**	0 (0)	11 (32)	.0008
**No mutations**	5 (16)	4 (12)	.500

Numbers in parenthesis are percentages. w- wild type sequence.

On multivariate analysis, which included all the parameters that achieved statistical significance in the univariate analysis (Table 2), only low disease-severity was found to be significantly associated with long-term remission. In patients with mild disease, the odds ratio for experiencing long-term remission was 4.95 (CI 1.31-18.64, P = 0.018) compared to patients with moderate to severe disease.

## Discussion

The data in the literature on periods of colchicine-free remission in FMF is scarce, therefore this disease trait is under-recognized [11-13]. To the best of our knowledge our study is the first to characterize this phenomenon. Compared to a regular FMF population, patients with long-term remission, occurring sometimes during the course of their disease, emerge as a unique group, with distinct clinical, demographic and molecular features. Clinically, this subset of patients has a mild disease, presenting mostly with abdominal attacks, and excellent response to low dose colchicine prevention. Demographically and genetically, this subset is distinct by the low rate of patients of North-African origin (a trend), and by absence of patients homozygous to the M694V mutation, both are markers of a severe phenotype [14-16]. The prevalence of disease remission in FMF population, which until currently was unknown, is estimated based on the present study as 3.3%. We believe that the findings of this study will help avoid the misinterpretation of long-term cessation of febrile episodes as an argument against the possibility of FMF.

Large proportion of patients (24%) related induction of remission to abdominal surgery. There is no data in the literature on this association, and this seemingly cause-and-effect relationship is still questionable. However, unawareness of such a relationship may lead to the erroneous misdiagnosis of FMF, as chronic recurrent appendicitis. In other words, recovery from a disease with recurrent episodes of abdominal pain, particularly after abdominal operations, should not be held as an argument against a diagnosis of FMF. Another association noted is between the flare of FMF that terminated the remission period and psychological stress, caused by major life events, such as becoming pregnant. Previous reports have also shown that stress (mental or physical), as well as pregnancy and menstrual cycle might trigger FMF attacks [12,13,17].

The delay in the diagnosis of FMF in patients with long-term remission led to a significant delay in the initiation of colchicine treatment. Nevertheless, only one of the patients in the remission group had clinical signs of chronic inflammation, such as amyloidosis, proteinuria or anemia. The laboratory finding that none of the patients in the remission group had elevated acute phase reactants commensurate with the absence of chronic sequelae. Thus, in patients with long term remission, the clinical and subclinical manifestations merge to reflect a subset of FMF with mild phenotype.

The unexpected insignificant difference in the proportion of patients of Northern-African origin between the remission and the control groups, is most likely due to the low rate of North-African patients in the control group (36%), reflecting a major demographic change in the FMF population, used to be dominated by subjects of North-African origin (53-61%) [13,18]. These demographic changes are marked by an increased proportion of FMF patients of European extraction and of mixed origin.

Long term remission is strongly determined by genetic factors, as reflected by the finding that none of the patients in the remission group was homozygous to the M694V mutation versus 32% in the control group. The absence of M694V homozygosity is consistent with the milder form of the disease and with the absence of amyloidosis in this group, as was shown previously [19]. On the other hand, the long term remission phenotype seems to also be affected by environmental factors, as implied from the finding that only one of 33 patients had siblings with long-term remission, despite multiplicity of additional family members with FMF in the study group.

The phenotype in the subset of patients with long-term remission is comparable to patients with late-onset FMF, published previously [20]. Both patient groups share low rates of extra-abdominal attacks and chronic manifestations, low disease severity, good response to colchicine and absence of homozygosity to the M694V mutation. These findings suggest that late-onset and long-term remission constitutes 2 faces of the same genetic-environmental make-up in FMF. Further subtyping of FMF into certain phenotypic classes may lead to increase understanding of the disease.

The main limitations of this cross sectional survey, stems from its dependence to some extent on patients’ recall of events. However, using computerized patient files, conducting a very thorough interview and carefully completing a detailed questionnaire should bridge this gap. Another limitation is the small sample size. However, this limitation is inevitable because remission in FMF is rare, and subjects of the study group were derived from a very large cohort of FMF patients, one of the largest worldwide. Thus, our findings seem to truly reflect the features of remission in FMF.

Third, an interventional study, looking for remission rate upon deliberate cessation of colchicine treatment in an unselected large cohort of FMF patients may yield higher than the 3.3% remission rate found by this observational study. However, obviously, such a study is unethical. Finally, the study neither tries to define patient candidature or appropriate time for colchicine termination, nor it explores long term outcomes of colchicine abstinence in FMF. These questions are beyond its scope. However, this proof of concept study establish and introduce the idea of colchicine free remission in FMF and features patients with such an experience, thus serving to incite future research in these directions.

## Conclusions

Long-term remission of FMF is an infrequent phenomenon that should be recognized by physicians treating FMF patients. FMF patients with long-term remission have distinct clinical, demographic and molecular characteristics allocating them to the very end of the mildest form of the disease.

## Competing interest

The authors declare that they have no competing interest.

## Authors’ contributions

AL, IBZ and TKV designed the study and wrote the manuscript. IBZ and TKV recruited patients to the study, collected the data and analyzed the results. OF, ML and SK recruited patients to the study and critically reviewed the manuscript. All authors read and approved the final manuscript.
